# Adipose mesenchymal stem cell transplantation alleviates spinal cord injury-induced neuroinflammation partly by suppressing the Jagged1/Notch pathway

**DOI:** 10.1186/s13287-020-01724-5

**Published:** 2020-06-03

**Authors:** Zhilai Zhou, Xiaobo Tian, Biling Mo, Huali Xu, Li Zhang, Lishan Huang, Shun Yao, Zixiang Huang, Yeyang Wang, Huan Xie, Liwei Xu, Hui Zhang

**Affiliations:** 1 The Spine Department, Orthopaedic Center, Guangdong Second Provincial General Hospital, Guangzhou, China; 2 The Second School of Clinical Medicine, Southern Medical University, Guangzhou, China; 3 Department of Cardiology, Liwan Central Hospital of Gaungzhou, Guangzhou, China; 4 Department of Anesthesiology, Zhu Jiang Hospital, Southern Medical University, Guangzhou, China

**Keywords:** Adipose, Mesenchymal stem cell, Spinal cord injury, Neuroinflammation, Jagged1, Notch

## Abstract

**Background:**

The therapeutic effects of adipose-derived mesenchymal stem cell (ADSC) transplantation have been demonstrated in several models of central nervous system (CNS) injury and are thought to involve the modulation of the inflammatory response. However, the exact underlying molecular mechanism is poorly understood. Activation of the Jagged1/Notch signaling pathway is thought to involve inflammatory and gliotic events in the CNS. Here, we elucidated the effect of ADSC transplantation on the inflammatory reaction after spinal cord injury (SCI) and the potential mechanism mediated by Jagged1/Notch signaling pathway suppression.

**Methods:**

To evaluate the therapeutic effects of ADSC treatment and the potential inhibitory effects of ADSCs on Notch signaling, mice were subjected to contusion SCI, and GFP-labeled ADSCs were injected into the lesion site immediately after the injury. Locomotor function, spinal cord tissue morphology, and the levels of Notch-related proteins and proinflammatory transcripts were compared between groups. To validate the hypothesis that the therapeutic effects of ADSCs are partly due to Notch1 signaling inhibition, a Jagged1 small interfering RNA (siRNA) was injected into the spinal cord to knock down Jagged1/Notch signaling. Neuronal staining and analyses of microglia/macrophage activation and signaling pathways were performed.

**Results:**

We demonstrated that ADSCs survived in the injured spinal cord for at least 28 days without differentiating into glial or neuronal elements. ADSC treatment resulted in significant downregulation of proinflammatory mediator expression and reduced ionized calcium-binding adapter molecule 1 (IBA1) and ED-1 staining in the injured spinal cord, eventually improving functional recovery. The augmentation of the Jagged1/Notch signaling pathway after SCI was suppressed by ADSC transplantation. The inhibition of the Jagged1/Notch signaling pathway by Jagged1 siRNA resulted in decreases in SCI-induced proinflammatory cytokines and the activation of microglia and an increase in the survival of neurons. Furthermore, Jagged1 knockdown suppressed the phosphorylation of JAK/STAT3 in astrocytes following SCI.

**Conclusion:**

The results of this study demonstrated that the therapeutic effects of ADSCs in SCI mice were partly due to Jagged1/Notch signaling pathway inhibition and a subsequent reduction in JAK/STAT3 phosphorylation in astrocytes.

## Introduction

Spinal cord injury (SCI) is one of the most challenging clinical issues, with an incidence of 15–40 cases per million people worldwide every year [1]. SCI induces a cascade of secondary tissue damage that limits spontaneous neural tissue regeneration, often leading to severe and permanent paralysis; despite great efforts, however, SCI repair remains a major therapeutic challenge for researchers [2]. Recent advances in SCI research have indicated that the neuroinflammatory process caused by injuries to the central nervous system (CNS), including the infiltration of macrophages into the injured tissue and the secretion of inflammatory cytokines, is one of the major causes of mortality and unfavorable outcomes in SCI patients [3, 4].

With the development of stem cell technology, the immunomodulatory function of stem cell transplantation, especially that of mesenchymal stem cells (MSCs), also known as mesenchymal stromal cells, has become a hot topic for the treatment of SCI [5]. MSCs have widely been used in experimental and clinical settings and have exhibited tangible therapeutic potential in various central nervous system (CNS) conditions, such as ischemic stroke [6, 7], multiple sclerosis [8], and SCI [9, 10]. It has been reported that MSC transplantation can reduce inflammatory reactions in pathological processes [11, 12]. MSCs were originally isolated from the bone marrow, and similar populations have been isolated from other tissues. Our previous study demonstrated that adipose tissue-derived mesenchymal stem cells (ADSCs) are a superior alternative to MSCs from other tissues because of their abundant availability and excellent expansion and proliferative capacities [13]. Numerous studies have demonstrated that ADSCs can alleviate the infiltration of ED1-positive macrophages and suppress the inflammatory response after CNS injury [14, 15]. However, the precise mechanism underlying the effects of transplanted MSCs on the inflammatory reaction following CNS is still unclear.

The Notch1 pathway is a highly conserved signaling system that is critical for cell fate decisions, tissue morphogenesis, and many cellular processes [16, 17]. The interaction of Notch receptors with their ligand (Delta1 or Jagged1) leads to the proteolytic cleavage of the transmembrane Notch receptor, giving rise to the release of the Notch intracellular domain (NICD) from the membrane, after which the NICD migrates into the nucleus. In the nucleus, NICD interacts with the transcription factor RBP-JK, which promotes the expression of transcriptional activators and thereby induces the expression of target genes, mainly hairy and enhancer of split (Hes) isoforms 1 and 5 [18, 19]. A growing body of work has demonstrated that Notch1 signaling is involved in the induction of the inflammatory response in various diseases. Pharmacological or transgenic interference with Notch signaling can ameliorate neuroinflammation and support neuronal survival [20–23]. In addition, MSC transplantation has been reported to suppress Notch1 signaling in various systemic diseases, such as ischemic stroke [24], inflammatory bowel disease [25], lupus [26], and brain injury [27]. However, the contributions of MSC administration to the inhibition of the Notch1 signaling pathway after SCI have not been explored.

Signal transducer and activator of transcription 3 (STAT3) is a critical regulator of astrogliosis and scar formation after CNS injury [28]. The activation of STAT3 via phosphorylation by Janus kinases (JAKs) has been demonstrated in a variety of disease models, including models of Alzheimer’s and Huntington’s diseases [29], cerebral ischemia [30], and SCI [31]. A large number of studies have indicated a strong association between Notch and JAK/STAT3 signaling, and a change in Notch expression can alter STAT3 phosphorylation and activity [32–34]. However, whether JAK/STAT3 signaling participates in the suppressive effects of MSCs on Notch1 expression after SCI is still unclear.

In this study, we investigated the spatiotemporal expression of Notch signaling in a mouse model of SCI and then elucidated the signaling pathways underlying the functional recovery of SCI mice treated with ADSC transplantation.

## Materials and methods

All the mice used in this experiment were purchased from the Laboratory Animal Center of the Southern Medical University (Guangzhou, China). All experimental protocols were approved by the Institutional Animal Care and Use Committee of Guangdong Second Provincial General Hospital, Guangzhou, China.

### Animals and experimental groups

The experiments were performed as described below.

#### Part 1

To evaluate the effects of ADSC treatment on the recovery of SCI mice and the activity of Notch1 signaling after SCI, a total of 132 8- to 12-week-old female C57Bl/6 mice were divided into three experimental groups: (i) the sham group, which underwent laminectomy without SCI; (ii) the control group, which underwent SCI followed by injection of PBS without cells; and (iii) the ADSC group, which underwent SCI followed by transplantation of ADSCs. The number of mice in each experimental group is listed in Additional file 1.

#### Part 2

To validate the hypothesis that the inhibition of the Jagged1/Notch signaling pathway participates in the protective mechanism of SCI, a lentiviral vector containing Jagged1 siRNA (Shanghai Lianfeng Biotechnology Co., Ltd., China) was injected into the spinal cord 72 h before SCI modeling to inhibit the Jagged1/Notch pathway. A total of 104 female C57Bl/6 mice were divided into four experimental groups: (i) the sham group; (ii) the SCI+PBS-injected (control) group; (iii) the SCI+Jagged1 siRNA-injected group, which received an injection of 2.5 μl of lentiviral vector (4 × 10^8^ IU/ml) 3 days before SCI; (iv) and the SCI+scramble siRNA-injected group, which was used as controls for the Jagged1 siRNA-injected group and received an injection of a lentiviral vector containing scramble siRNA. The number of mice in each experimental group is listed in Additional file 1.

### Preparation of ADSCs

ADSCs were isolated as previously described [35]. Adipose tissues from the inguinal pads were dissected, and upon the removal of debris, the tissues were then enzymatically dissociated with 0.1% collagenase type I (Sigma, St. Louis, MO, USA) at 37 °C for 60 min under shaking. The digested tissue was centrifuged at 1500 rpm for 5 min and resuspended in saline for a total of 2 times. Isolated cells were resuspended in Dulbecco’s modified Eagle’s medium (DMEM; Gibco, Grand Island, NY, USA) with 10% fetal bovine serum (Gibco) and maintained at 37 °C in 5% CO_2_. Following overnight incubation, the flasks were washed extensively with PBS to remove non-adherent cells. Fluorescence-activated cell sorting analysis was used to identify the phenotype of the cells. CD29, CD44, CD90, and CD45 were detected to confirm the MSC identity of the cells. ADSCs at passage 3 were used in this study. To track the grafted cells in vivo, ADSCs were infected with GFP-expressing lentivirus as described previously [36]. Transduction efficiency was assessed by the detection of the GFP signal intensity. GFP-modified ADSCs were detached with 0.125% (w/v) trypsin, suspended in phosphate-buffered saline (PBS), and kept on ice until use.

### Primary neuron culture, OGD stress model, and co-culture systems

Neurons were cultured from the cerebral cortices of neonatal mice brains (day 1). Cultures were prepared according to a previously described procedure with some modifications [37]. Briefly, the cerebral cortex was dissociated in Ca^2+^ and Mg^2+^ free Hank’s Balanced Saline Solution (Life Technologies; Grand Island, NY, USA) containing 0.2% trypsin for 15 min. The cell suspensions were washed and resuspended with Dulbecco’s modified Eagle’s medium (DMEM; Gibco) with 5% fetal bovine serum (FBS; Gibco). The neurons were seeded into poly-l-lysine precoated 24-well plates in a density of 10^4^ cells/well. Following cell attachment (3–6 h after plating), the culture medium was replaced with a neurobasal medium (Invitrogen, Waltham, MA, USA) containing 2% B-27 (Gibco). Three days later, the neuron culture medium was replaced with Hank’s Balanced Salt Solution. Then, the cells were placed in an incubator (Thermo Forma 3111, Thermo Scientific) containing 5% O_2_ and 95% N_2_ at 37 °C for 6 h to establish an oxygen and glucose deprivation (OGD) model. Neurons that were not subjected to OGD served as controls. For generating a co-culture system, 2 × 10^6^ ADSC were seeded together with the OGD neurons in a total of 0.5 mL media.

### In vitro Jagged-1 Fc and Jagged-1 siRNA treatments

To activate Jagged-1/Notch signaling, ADSC and neurons were co-cultured in 0.5 ml DMED containing 1 μg/ml Jagged-1 Fc (Abcam, Cambridge, MA, USA) for 24 h. To block Jagged-1/Notch signaling, neurons were transfected with Jagged-1 siRNA for 24 h according to the manufacturer’s protocol, and then, these cells were co-cultured with ADSC for 24 h.

### Enzyme-linked immunosorbent assay

The levels of IL-6, IL-1β, and TNF-α cytokine release in the culture media from the various treatment groups were measured using an ELISA kit (R&D, Minneapolis, MN, USA). The colorimetric absorbance was recorded at a wavelength of 450 nm. The IL-6, IL-1β, and TNF-α concentrations were calculated according to a standard curve constructed for each assay, and each assay was performed in triplicate.

### SCI model and ADSC transplantation

After anesthetization by inhalation of 1.5% isoflurane, the animals underwent dorsal laminectomy at the tenth thoracic vertebral level (T10), and the spinal cord and dura mater were exposed. A moderate contusion injury (~ 50 kdyn) of the spinal cord was produced using the Infinite Horizon Impactor (Precision Systems and Instrumentation) as previously described [38]. After injury, depending on the experimental group, 3 μl of saline- or GFP-labeled ADSCs (1 × 10^6^ cells) was injected directly into the SCI epicenter with a Hamilton syringe positioned with a stereotaxic instrument (Kopf Instruments, Tujunga, CA, USA) at a flow rate of 0.4 μl/min. The overlying muscle layers were then sutured, and the cutaneous layers were stapled. The bladders of the mice were manually and gently expressed to prevent urinary tract infection until the reflexive control of micturition was restored. Depending on the experiment performed, the SCI mice were killed by transcardial perfusion 1, 3, 7, 14, or 28 days post-contusion.

### Behavioral analyses

Functional recovery after SCI was assessed with the Basso Mouse Scale (BMS) scores [39]. Mice were tested on postoperative days 1, 7, 14, 21, and 28 for the duration of the experiments. All animals were placed in an open field and simultaneously observed by two examiners who were blinded to the identity of the animals.

### Western blot analysis

According to experimental design, mice (*n* = 4) were transcardially perfused with ice-cold PBS (0.1 M, pH 7.4), and approximately 5 mm of each spinal cord segment, including the injury site, was collected and stored at − 80 °C until further analysis. Equal amounts of protein (50 μg) from different samples were separated by sodium dodecyl sulfate/polyacrylamide gel electrophoresis and transferred to nitrocellulose membranes. The blots were incubated with the following primary antibodies: Notch1 (mouse, 1:1000, Santa Cruz Biotechnology, Dallas, TX, USA), RBP-JK (mouse, 1:1000, Santa Cruz Biotechnology), NICD (rabbit, 1:2000, Abcam, Cambridge, MA, USA), JAK (rabbit, 1:2000, Cell Signaling Technology, Inc., Beverly, MA, USA), p-STAT3 (rabbit, 1:2000, Cell Signaling Technology), and STAT3 (rabbit, 1:2000, Cell Signaling Technology). The primary antibodies were further incubated with appropriate secondary antibodies (1:10,000) for 2 h at room temperature. GAPDH was used as an internal control (Sigma, St. Louis, MO, USA). The reactive bands were visualized using enhanced chemiluminescence reagent (Thermo Fisher Scientific, Waltham, USA), and the density of the protein bands was semiquantified using The ImageJ software.

### Real-time polymerase chain reaction

RNA was isolated using TRIzol Reagent (TaKaRa Biotechnology, Dalian, China), and reverse transcripts were synthesized from 1 μg of total RNA using the PrimeScript 1st Strand cDNA Synthesis Kit (TaKaRa Biotechnology, Dalian, China) according to the manufacturer’s instructions. Amplification was performed using the ABI 7500 Real-Time PCR System (Applied Biosystems, Foster City, CA, USA) with a two-step PCR protocol (preincubation of 10 min at 95 °C followed by 30 cycles at 95 °C for 15 s and 60 °C for 1 min). The list of primer sequences is provided in Table 1. After normalization to β-actin mRNA, the comparative threshold (ΔCT) method was used to examine the relative quantification of the samples (Relative Quantitation computer software; Applied Biosystems). Fold expression changes were calculated using the equation 2ΔΔCT.

**Table 1:** Primers for real-time PCR

Gene	Sequence
IL-6	GAGTCCTTCAGAGAGATACAGAAAC
	TGGTCTTGGTCCTTAGCCAC
IL-1β	GAAATGCCACCTTTTGACAGTG
	CTGGATGCTCTCATCAGGACA
TNF-α	CCCTCACACTCAGATCATCTTCT
	GCTACGACGTGGGCTACAG
β-actin	ATCAACGACCCCTTCATTGACC
	CCAGTAGACTCCACGACATACTCAG

### Hematoxylin and eosin staining

We performed hematoxylin and eosin (H&E) staining to examine the lesion areas in the injured spinal cords of the control group and ADSC group at 28 days post-injury (*n* = 4). H&E staining was performed in accordance with a well-established protocol. After curing of the mounting medium, the sections were examined under a light microscope (Zeiss, Germany). The size of the cavity formed at the injury epicenter was manually outlined for each section and analyzed using the ImageJ software.

### Immunostaining

The mice were anesthetized and transcardially perfused with 4% paraformaldehyde in PBS on days 1, 3, 7, 14, and 28 after SCI. The spinal cords were post-fixed in paraformaldehyde overnight, embedded in paraffin, sectioned in the sagittal or transverse plane at a thickness of 4 μm, and deparaffinized and rehydrated by subsequent immersion in xylene (2 times, 10 min), 100% ethanol (5 times, 1 min), 95% ethanol (5 min), 90% ethanol (3 min), 80% ethanol (3 min), 70% ethanol (3 min), 80% ethanol (5 min), and H_2_O (5 min). The following primary antibodies were used: GFP (1:50, mouse, Santa Cruz Biotechnology), Jagged1 (mouse, 1:50, Santa Cruz Biotechnology), NICD (rabbit, 1:200, Cell Signaling Technology), GFAP (mouse, 1:1000, Sigma-Aldrich, Shanghai; rabbit, 1:1000, Millipore, Billerica, MA, USA), IBA1 (rabbit, 1:1000, Abcam), NeuN (1:1000, mouse, Millipore; 1:100, rabbit, Beyotime, Bejing, China), NF200 (rabbit, 1:800, Abcam), ED1 (mouse, 1:200, Millipore), activated caspase-3 (rabbit, 1:100, Cell Signaling Technology), and pSTAT3 (rabbit, 1:200, Cell Signaling Technology). Antigen retrieval was performed with sodium citrate buffer (10 mM sodium citrate, pH 6, 0.01% Triton) for 20 min at 96 °C in a microwave. For immunostaining with antibodies against Jagged1 (mouse, 1:50, Santa Cruz Biotechnology) and NICD (rabbit, 1:200, Cell Signaling Technology), the mounted sections were subjected to sodium citrate buffer-based (10 mM sodium citrate, pH 6, in 0.01% Triton) antigen retrieval in a preheated pressure cooker for 5 min. The secondary antibodies (1:500) that were used were Alexa Fluor 594-conjugated goat anti-rabbit (Thermo Fisher, Waltham, MA, USA) and Alexa Fluor 488-conjugated goat anti-mouse (Thermo Fisher). Finally, the tissues were counterstained with 4′6-diamidino-2-phenylindole (DAPI) (Sigma, St. Louis, MO, USA) and observed under a fluorescence microscope (Leica-DMI8, Leica Microsystems, Germany).

### Quantitative analyses of stained tissue sections

To quantify cells of interest in immunostained sections, we obtained images of the stained sections by fluorescence microscopy (model TCS SP2; Leica Microsystems). Three slices per mouse and three fields from each slice were used for quantification; we manually outlined the cells and quantified them using the ImageJ software. The threshold values were maintained at a constant level for all analyses using the ImageJ software. ED1-positive, pSTAT3-positive, IBA1-positive, and NICD-positive areas were divided by the area of the image and are expressed as percentages. The number of NeuN-positive cells and activated caspase 3-positive cells was manually counted at × 400 magnification. The average number of activated caspase 3-positive cells expressing NeuN in these images was quantified.

### Statistical analysis

Statistical analysis was performed using SPSS 20.0 for Windows (Inc., Chicago, IL, USA). All data in this study are presented as the mean ± SEM. Data from more than 2 groups were analyzed by one-way analysis of variance (ANOVA), followed by Bonferroni post hoc testing. The differences between the 2 groups were analyzed by Student’s *t* tests. Differences were deemed statistically significant at *P* < 0.05.

## Results

### ADSC treatment improved functional recovery after SCI

**Fig. 1:**
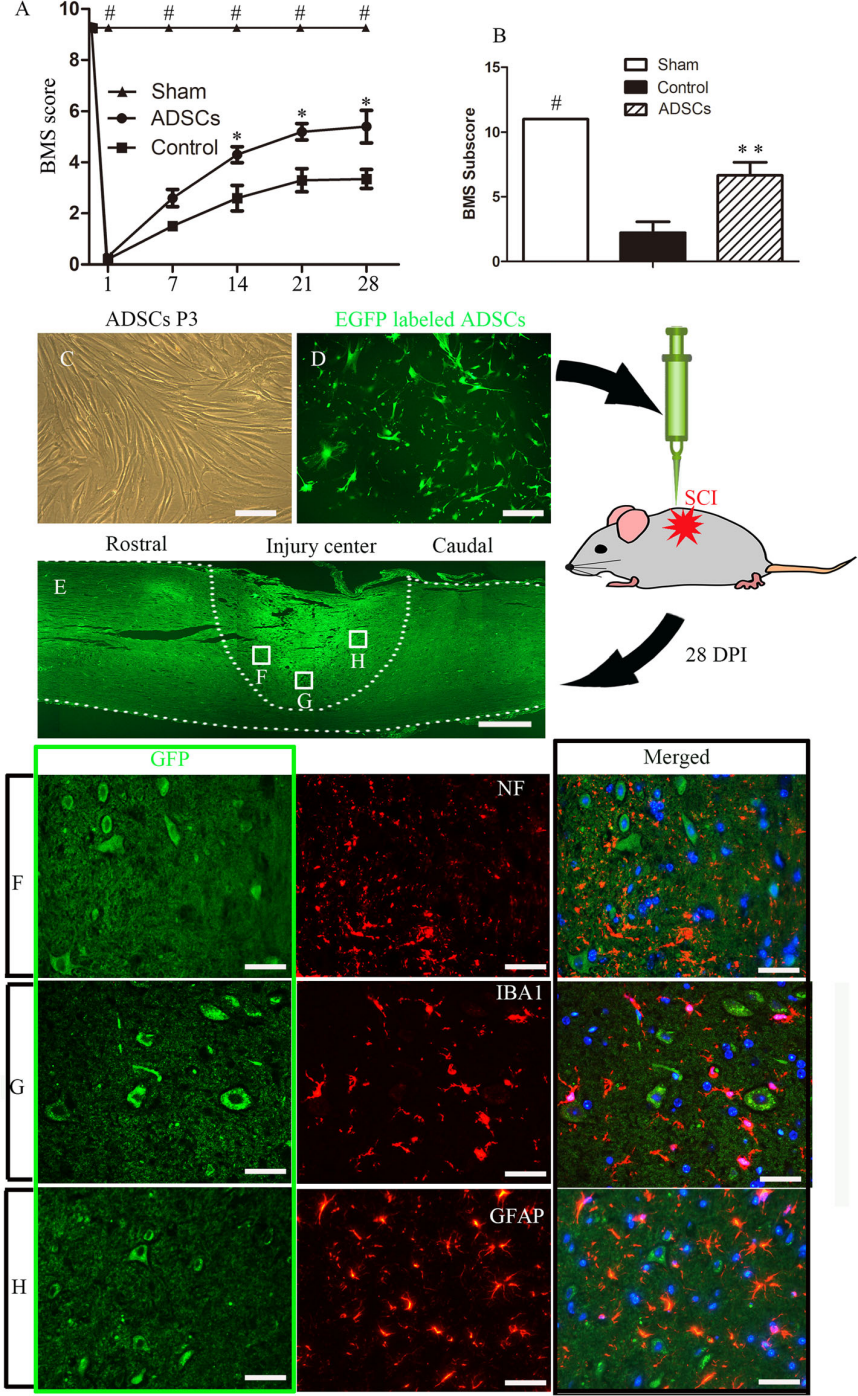
Transplanted ADSCs survived in the injured spinal cord in a mouse model of SCI. ADSC treatment promoted the recovery of the locomotor capacity of SCI mice, as evaluated by primary BMS scores (**a**) at different time points and terminal BMS subscores (**b**) (*P < 0.05 versus the control group; **P < 0.01 versus the control group; ^#^*P* < 0.05 versus the other two groups). **c** The morphology of ADSCs in cell culture at passage 3. Cultured cells showed typically spindle-shaped morphology under phase-contrast microscopy. **d** ADSCs infected with GFP reporter genes showed strong fluorescent staining. **e** A horizontal section of the spinal cord from the ADSC group containing GFP-positive donor cells at the injury site. Double immunofluorescence staining showed that GFP-positive donor cells were negative for the neuronal protein NF200 (**f**), the microglial marker IBA1 (**g**), and the glial protein GFAP (**h**). Scale bars = 100 μm (**c**, **d**); scale bar = 200 μm (**e**); scale bars = 50 μm (**f**–**h**)

Locomotor functional recovery was evaluated using the Basso, Beattie, Bresnahan-derived Basso Mouse Scale (BMS) locomotor rating scale scores, which included primary scores and subscores, 1 day before the injury as well as 1, 7, 14, 21, and 28 days after SCI. The scores of the control group increased gradually and reached a plateau at approximately 3 weeks. Significant increases in the BMS scores of SCI mice treated with ADSCs compared to mice in the PBS control group were found beginning on day 14, and these increases continued until the end of the observation on day 28 (*P* < 0.05, Fig. 1a). Furthermore, our data revealed that ADSC treatment was associated with significantly higher terminal BMS subscores (*P* < 0.05, Fig. 1b). The BMS scores in the sham group were significantly higher than those in the PBS control group and ADSC group at any time points. These data demonstrate that ADSC transplantation can significantly ameliorate the functional deficits generated in this mouse model of SCI.

### Transplanted ADSCs survived in the injured spinal cord

Next, we performed immunohistochemical staining to determine the fate of the transplanted cells. The characterization of ADSCs at passage 3 is shown in Fig. 1c.

After they were transduced with a lentiviral vector encoding the GFP reporter gene, most of the ADSCs expressed strong green fluorescence under fluorescence microscopy (Fig. 1d). Four weeks after the transplantation of ADSCs, most of the GFP-positive cells were located around the center of the lesion site (Fig. 1e). No GFP-positive cells were detected in the PBS control group (data not shown). In addition, to evaluate the neural differentiation potential of ADSCs in the spinal cord environment, the expression of neuronal and glial marker proteins was immunohistochemically analyzed 4 weeks after SCI. Double fluorescence immunohistochemistry revealed that ADSCs failed to express the neuronal marker neurofilament, the glial protein GFAP, and the microglial marker IBA1 4 weeks after grafting (Fig. 1f–h). ADSCs did not obviously differentiate into neurons, astrocytes, or microglia after transplantation into the acutely injured spinal cords.

### ADSC administration inhibited the infiltration of macrophages and reduced the expression of inflammatory cytokines following SCI

**Fig. 2:**
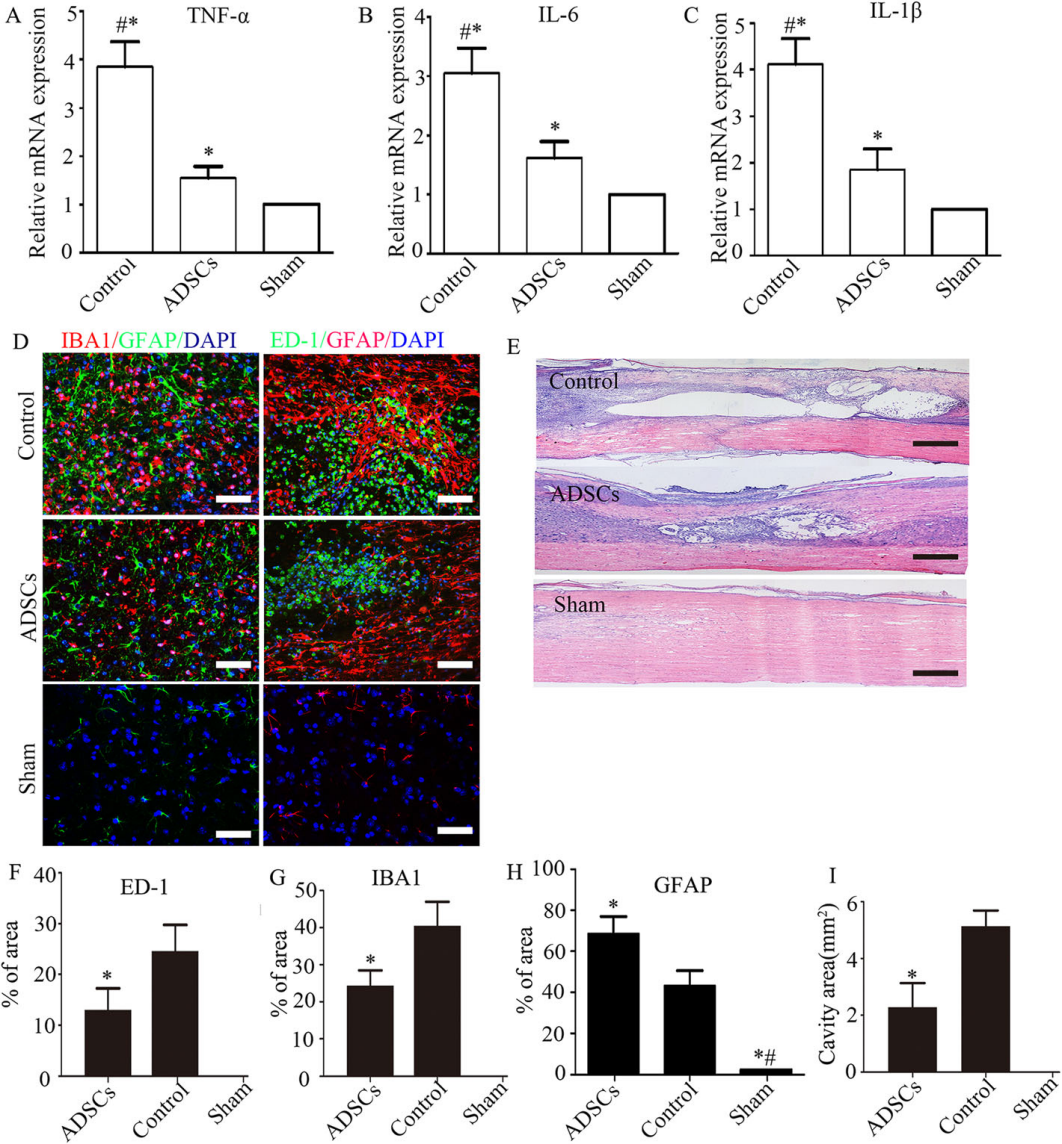
ADSC administration suppressed the inflammatory response following SCI. ADSC treatment decreased the expression of the proinflammatory cytokines TNF-α (**a**), IL-6 (**b**), and IL-1β (**c**) in the spinal cord tissue at 3 days post-SCI (*n* = 5; the data are expressed as the mean ± SD; *P < 0.05 versus the Sham group, ^#^*P* < 0.05 versus the ADSC group). Representative images of double immunohistochemical staining of IBA1 and GFAP (**d**) and of ED-1 and GFAP (**f**) in different groups. Representative images were taken at the boundary of the injury. **e** Histological images (H&E staining) of longitudinal sections of the uninjured spinal cord and spinal cords at 28 days post-SCI with different treatments. The quantification of the ED-1-positive (**f**), IBA1-positive (**g**), and GFAP-positive (**h**) areas (*n* = 4; the data are expressed as the mean ± SD; *P < 0.05 versus the control group, ^#^*P* < 0.05 versus the ADSC group). **i** The cavity area calculated based on H&E staining (*n* = 4; the data are expressed as the mean ± SD; *P < 0.05 versus the control group). Scale bars = 50 μm (**d**, **f**); scale bars = 200 μm (**e**)

Considering that neural regeneration requires a permissive immune microenvironment, we then explored the effects of ADSC treatment on macrophage/microglia-mediated inflammation after SCI. We first examined the mRNA expression of proinflammatory signals, including TNFα, IL-1β, and IL-6, 3 days after injury. Our data demonstrated that the mRNA levels of TNFα, IL-1β, and IL-6 were significantly upregulated in the SCI group. Compared with the SCI group, the mRNA levels of TNFα, IL-1β, and IL-6 were decreased by 55.61 ± 3.22%, 48.96 ± 3.31%, and 42.64 ± 3.84%, respectively, in the ADSC group (all *P* < 0.05) (Fig. 2a–c). Meanwhile, the relative fluorescence intensity of the inflammatory infiltrates was analyzed 28 days after injury. In the present study, ED-1 was used as a marker of the activation of resident microglia and extravasated macrophages, while IBA1 was used as a marker of total microglia. We found that the ADSC group exhibited significantly fewer areas of IBA1-positive, ED-1-positive, and GFAP-positive cells than the control group (all *P* < 0.05) (Fig. 2d, f–h). No IBA1-positive and ED-1-positive cells were observed in the spinal cords of sham-operated mice (Fig. 2d). The change in cavity size in the sham group, control group, and ADSC group was also measured. H&E staining of tissue samples showed a significantly reduced cavity size in the ADSC group compared with the control group (*P < 0.05) (Fig. 2e, i).

### The Notch signaling pathway was activated in the spinal cords of SCI mice

**Fig. 3:**
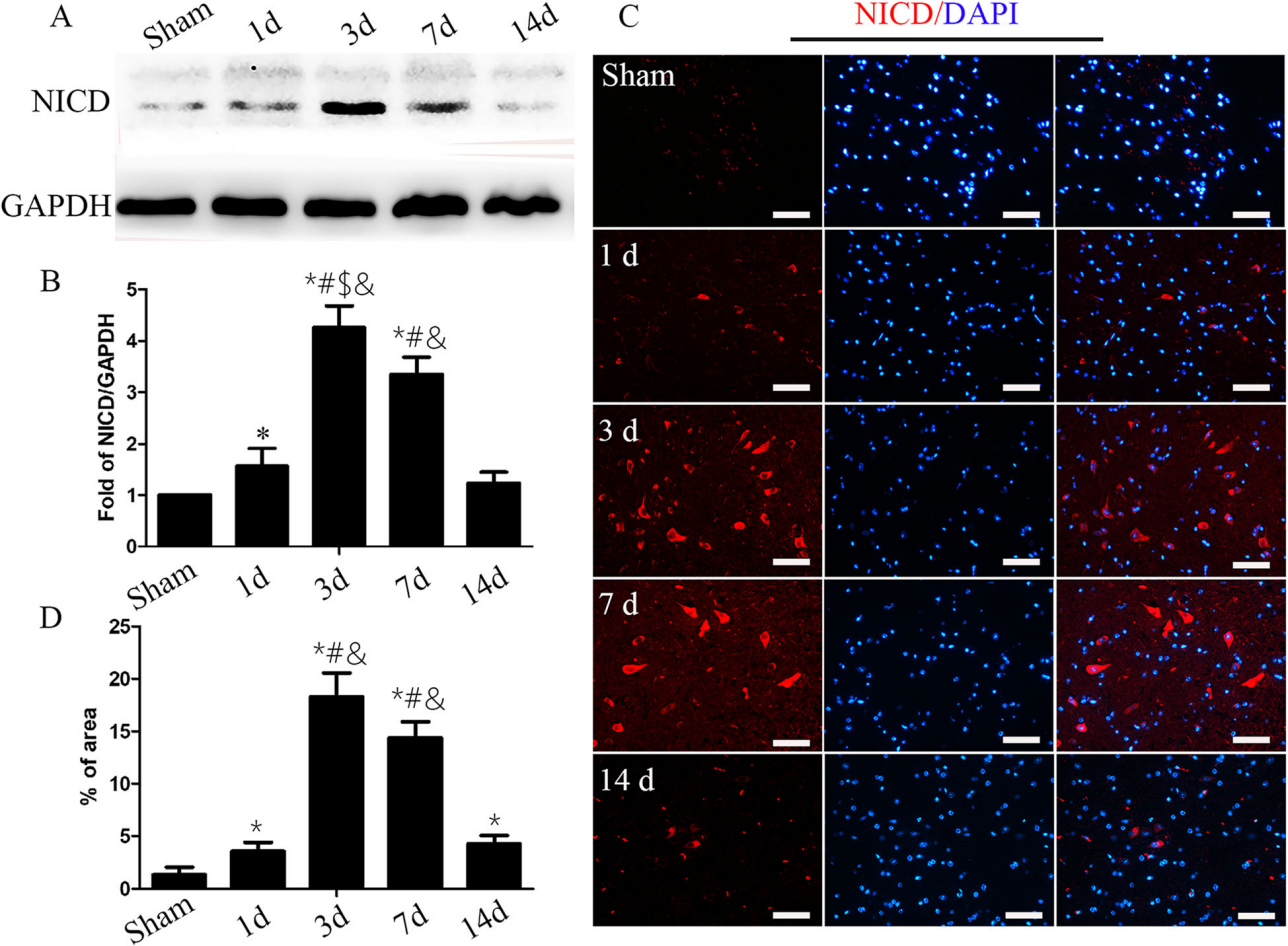
The Notch pathway was activated after SCI. **a** Western blot analysis showing that NICD expression began to increase 1 day after SCI and reached its peak expression 3 days after SCI. GAPDH was used as a loading control. **b** The quantification of NICD expression (*n* = 4 in each group; the data are expressed as the mean ± SEM; *P < 0.05 versus the sham group; ^#^*P* < 0.05 versus 1 day; ^$^*P* < 0.05 versus 7 days; ^&^*P* < 0.05 versus 14 days). **c** Immunostaining of NICD before and 1, 3, 7, and 14 days after SCI. Scale bar = 50 mm. **d** The quantification of NICD fluorescence intensity (*n* = 4 in each group; the data are expressed as the mean ± SEM; *P < 0.05 versus the sham group; ^#^*P* < 0.05 versus 1 day; ^&^*P* < 0.05 versus 14 days)

To examine the activation of Notch signaling after SCI, we determined the expression profile of NICD, the activated form of Notch1, in the spinal cords of SCI mice (1, 3, 7, and 14 days after injury) and sham mice by western blotting. We found that the NICD expression in the spinal cords of SCI mice began 1 day after the injury, reached a peak at 3 days, and then gradually decreased to baseline at 14 days (Fig. 3a, b). Immunocytochemistry was used to further confirm the expression pattern of NICD, as shown in Fig. 3c. Three days after SCI, NICD staining was predominantly found in the ventral horn of the spinal cord. The intensity of NICD was significantly elevated 3 days after SCI (versus the sham group and at 1 day, *P* < 0.05) and decreased 7 days and 14 days later (Fig. 3c, d). Collectively, these results suggest that Notch1 signaling is activated after SCI.

### Neurons were the cells involved in Notch signaling in SCI mice

**Fig. 4:**
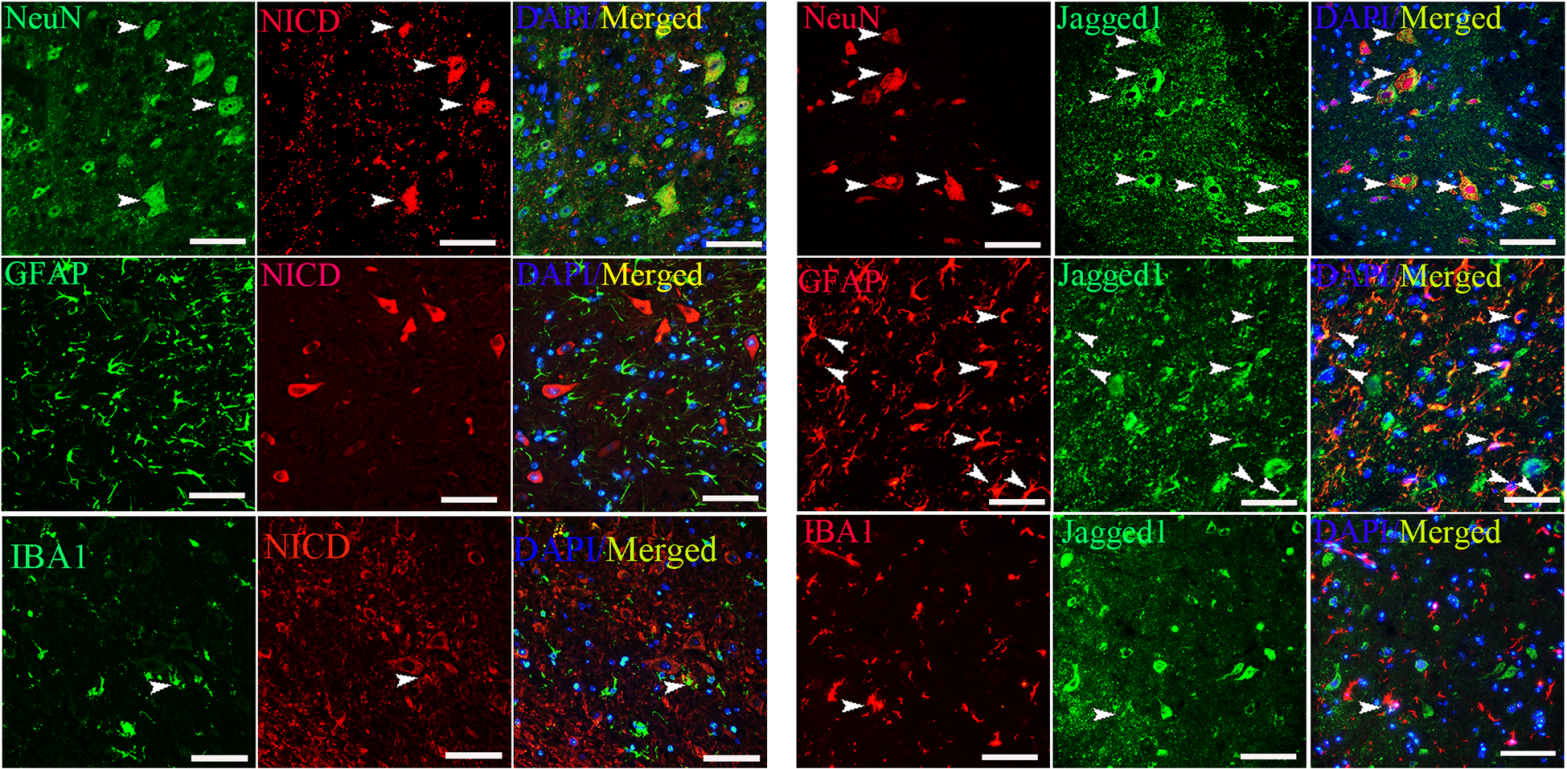
Neurons were the main cells that mediated Notch signaling after SCI. Double immunofluorescence staining of NICD and Jagged1 in the spinal cords of the SCI group mice 7 days after SCI. The nuclei were stained with DAPI (blue). NICD was mainly localized in NeuN-positive neurons, no NICD signal was observed in astrocytes, and only a small portion of IBA1-positive microglia/macrophages were positive for NICD. Jagged1 was obviously expressed by NeuN-positive neurons and GFAP-positive astrocytes, and the Jagged1 signal in IBA1-positive microglia/macrophages was weak. Scale bars: 50 μm

To confirm which cell types mediate Notch signaling in the spinal cord, we determined the cellular localization of NICD and Jagged-1 using double immunofluorescence assays. Our results showed that NICD was predominantly localized to NeuN-positive neurons, only a small portion of the IBA1-positive macrophages/microglia were positive for NICD, and no NICD signal was observed in GFAP-positive astrocytes. Jagged1 was abundantly expressed in GFAP-positive astrocytes and NeuN-positive neurons. The expression of Jagged1 in IBA1-positive macrophages/microglia was hardly detectable (Fig. 4). Collectively, these results indicate that neurons are the predominant cells involved in Notch signaling activation after SCI.

### ADSC treatment inhibited the activation of the Jagged1/Notch signaling pathway

**Fig. 5:**
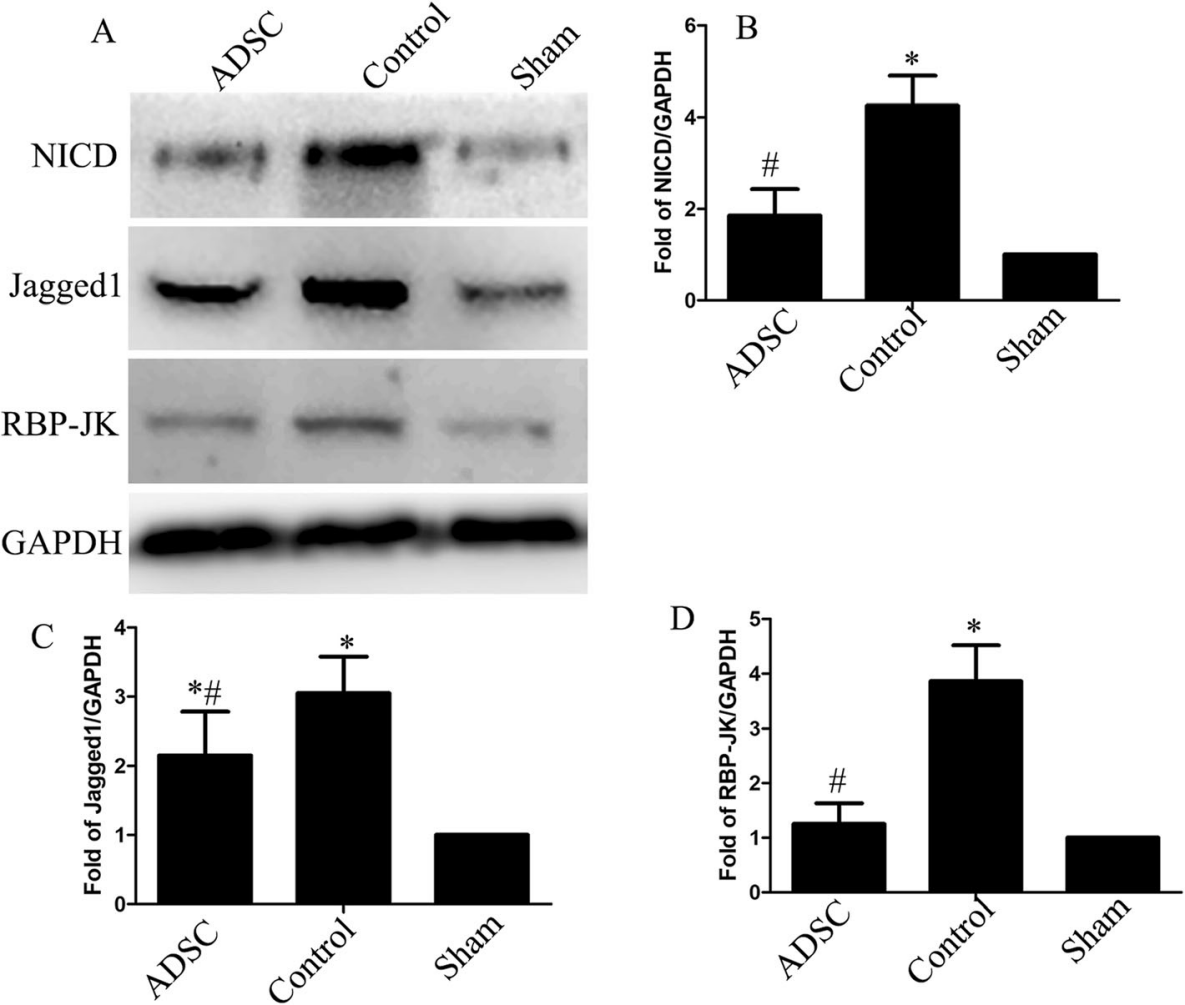
ADSC transplantation inhibited the SCI-induced upregulation of Notch1 pathway-related molecules. **a** Representative western blots of Notch1 signaling pathway-associated proteins after ADSC treatment at 3 days post-injury and in the uninjured spinal cords. The quantification of the relative expression levels of NICD (**b**), Jagged1 (**c**), and RBP-JK (**d**) normalized to the level of GAPDH (*n* = 4; the data are expressed as the mean ± SEM; *P < 0.05 versus the sham group; ^#^*P* < 0.05 versus the control group)

To explore the underlying mechanism of ADSC treatment in SCI mice, we performed western blotting to evaluate the expression of Notch1 signaling pathway-associated factors 3 days after ADSC treatment. The western blotting results demonstrated that SCI increased the NICD, Jagged1, and RBP-JK expression and that this increase was attenuated after ADSC transplantation (Fig. 5a–d).

### Knockdown of the Jagged1/Notch signaling pathway by Jagged1 siRNA decreased the inflammatory response and protected neuronal integrity

**Fig. 6:**
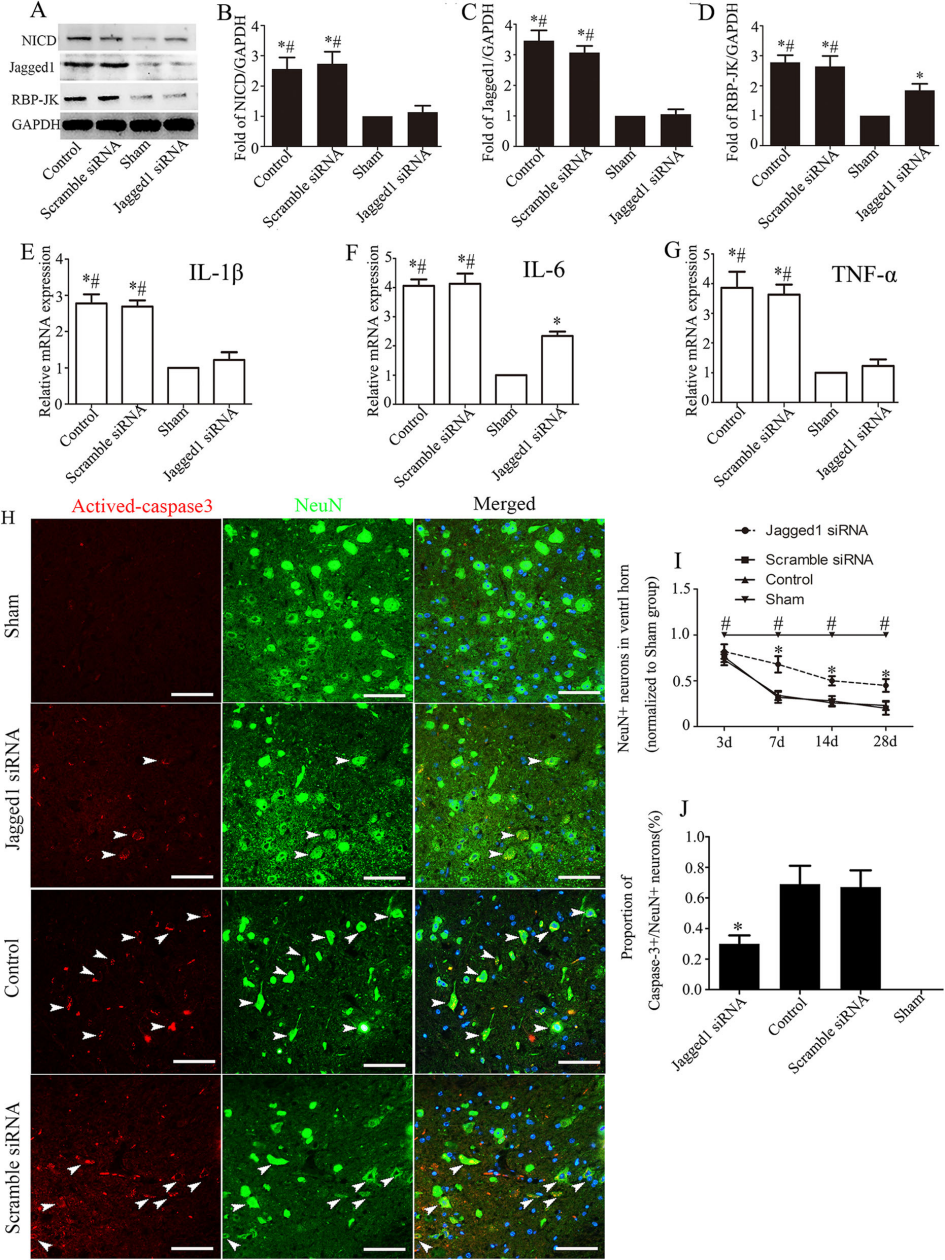
Jagged1/Notch signaling knockdown reduced SCI-induced neuroinflammation and promoted neuronal preservation. **a** Representative western blots of Notch1 signaling pathway-associated proteins at 6 days after Jagged1 siRNA injection (3 days after SCI) and in the uninjured spinal cords. The quantification of the relative expression levels of NICD (**b**), Jagged1 (**c**), and RBP-JK (**d**) normalized to the level of GAPDH (*n* = 4; the data are expressed as the mean ± SEM; *P < 0.05 versus the sham group; ^#^*P* < 0.05 versus the SCI+Jagged1 siRNA-injected group). Jagged1 siRNA injection decreased the expression of the proinflammatory cytokines IL-1β (**e**), IL-6 (**f**), and TNF-α (**g**) in the spinal cord tissue at 3 days post-SCI (*n* = 4; the data are expressed as the mean ± SD; *P < 0.05 versus the sham group; ^#^*P* < 0.05 versus the SCI+Jagged1 siRNA-injected group). **h** Representative images of activated caspase-3/NeuN staining neurons in various groups. **i** The relative number of intact neurons showing strong NeuN staining at 3, 7, 14, and 28 days after SCI (*n* = 4; the data are expressed as the mean ± SEM; *P < 0.05 versus the SCI+scramble siRNA-injected mice and the SCI control group, ^#^*P* < 0.05 versus the other groups). **j** The proportion of NeuN-positive neurons that were also positive for activated caspase-3 (*n* = 4; *P < 0.05 versus the other groups)

To confirm the functional implications of Jagged1/Notch1 activation after SCI, Jagged1 expression was knocked down by injecting a lentivirus containing Jagged1 siRNA into the mouse spinal cord 3 days before SCI modeling. The inflammatory response after SCI was subsequently observed. The western blotting results showed that the protein levels of NICD, Jagged1, and RBP-JK were significantly reduced in the mouse spinal cord 6 days after Jagged1 siRNA injection (3 days after SCI) (*P* < 0.05, Fig. 6a–d). We next examined the effect of Jagged1/Notch1 knockdown on the inflammatory response. As shown in Fig. 6e–g, q-PCR examination suggested that Jagged1 siRNA injection significantly decreased the SCI-induced upregulation of the proinflammatory cytokines IL-1β (a), IL-6 (f), and TNF-α (g) at 6 days after Jagged1 siRNA injection (3 days after SCI). The proinflammatory response was always accompanied by neuronal death after SCI. To obtain direct evidence that Jagged1/Notch1 inhibition contributes to neuronal preservation, we further performed NeuN staining to detect surviving neurons in the spinal cord 3, 7, 14, and 28 days after SCI. Viable neurons were defined by strong NeuN staining. Our results showed that from day 7, the preservation of neuronal integrity in the SCI+Jagged1 siRNA-injected group was significantly increased compared to that in the control group and SCI+scramble siRNA-injected group, while there was no difference between the control group and the SCI+scramble siRNA-injected group (Fig. 6i). Neuronal death was confirmed by activated caspase-3/NeuN co-labeling at 7 days after SCI. In the sham group, activated caspase-3 immunoreactivity was undetectable. In the spinal cords of SCI control mice and SCI+scramble siRNA-injected mice, activated caspase-3 immunoreactivity was exhibited in 62.42 ± 3.44% and 68.48 ± 5.64% of neurons, respectively, which was almost twice as high as the percentage of activated caspase-3-positive neurons observed in the spinal cords of SCI+Jagged1 siRNA-injected mice (Fig. 6h, j).

### ADSC protected against Jagged1/Notch pathway-mediated inflammation in neurons subjected to OGD injury

The in vivo data suggest that ADSC transplantation suppressed the Jagged1/Notch pathway and alleviated the SCI-induced neuroinflammation, neurons were the main cells involved in Notch signaling in SCI mice. We also performed in vitro studies to confirm and further evaluate these results. Concentrations of IL-6, IL-1β, and TNF-α in conditioned media from neurons with different treatments were measured by ELISA. Our results show the concentration of IL-6 from neurons alone was below the detectable level even after they had been exposed to OGD stress for 24 h (data were not shown). The level of TNF-α and IL-1β secreted from neurons following OGD exposure was significantly increased by more than 3-fold compared with that of non-OGD exposed cells (*P* < 0.05) (Supplementary Fig. 1A, B). ADSC co-culture significantly suppressed the release of TNF-α and IL-1β compared with that in monocultured OGD-injured neurons (Supplementary Fig. 1A, B). The suppressive effect was further enhanced by Jagged1 siRNA treatment, whereas Jagged1 Fc treatment abolished these suppressive effects. Furthermore, we used western blotting to measure the expression of activated caspase3 in OGD-injured neurons after Jagged1 siRNA or Jagged1 Fc treatment. The changes in the protein expression levels of activated caspase3 mirrored those of TNF-α and IL-1β following Jagged1 siRNA or Jagged1 Fc treatment (Supplementary Fig. 1C, D).

### The JAK/STAT3 pathway is repressed by the inhibition of Jagged1

**Fig. 7:**
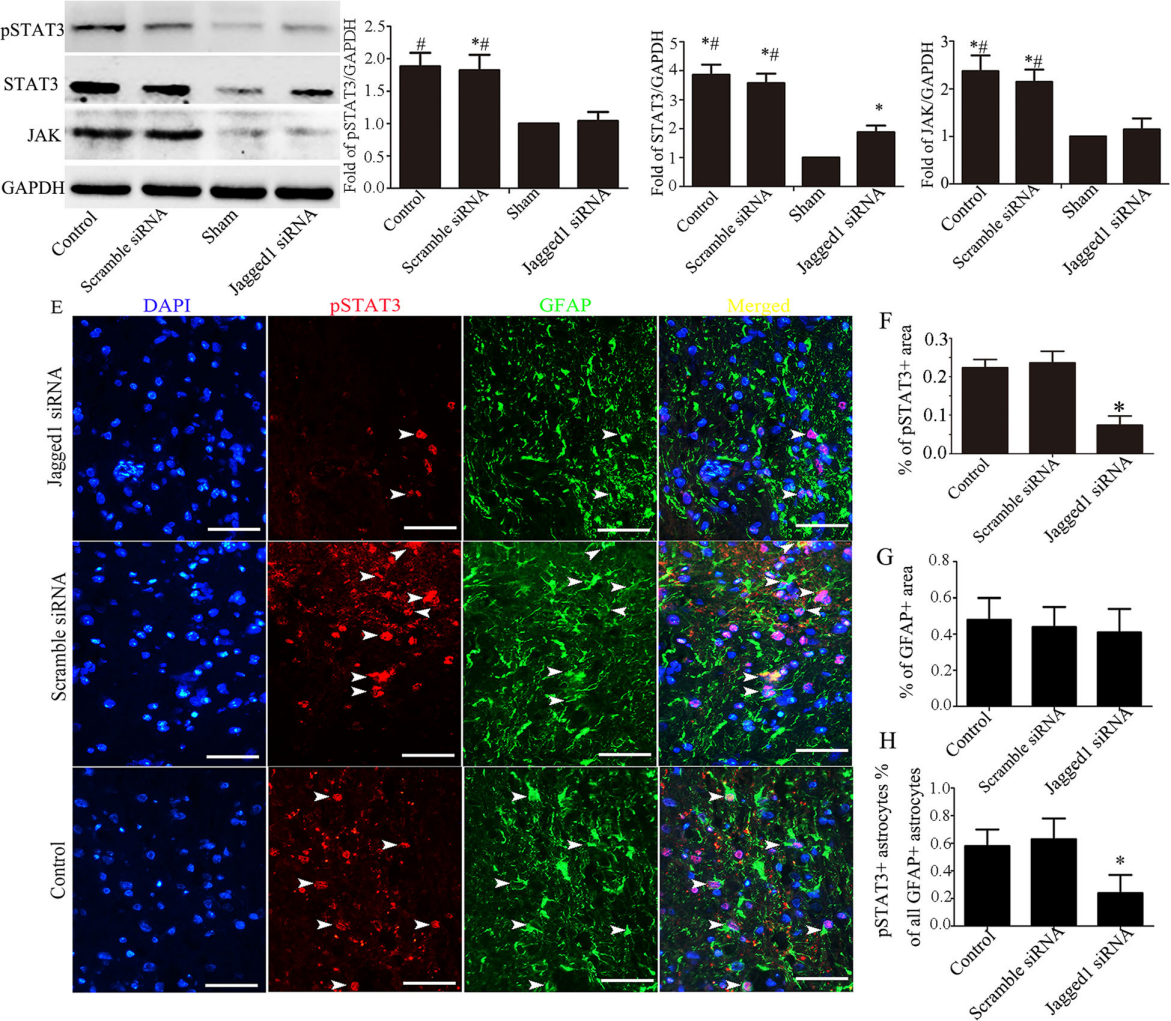
Jagged1 knockdown inhibited JAK/STAT3 signaling in astrocytes. **a** Representative western blot images showing the protein levels of JAK/STAT3 pathway-related molecules at 3 days after Jagged1 siRNA injection. The quantification of p-STAT3 (**b**), STAT3 (**c**), and JAK (**d**) (*n* = 4; the data are expressed as the mean ± SEM; *P < 0.05 versus the sham group; ^#^*P* < 0.05 versus the SCI+scramble siRNA group). **e** Representative images of the lesion area in the spinal cord sections double-stained for pSTAT3 and GFAP. The cell nuclei were counterstained with DAPI. **f** The quantification of the STAT3-positive area. Jagged1 siRNA injection significantly reduced the STAT3-positive area at 3 days after SCI. **g** The quantification of the GFAP-positive area. There was no significant difference in the GFAP-positive area among the three groups at 3 days after SCI

The activation of the JAK/STAT3 pathway has been observed to be correlated with astrocyte reactivity in several types of acute injury. Several reports have shown that there is crosstalk between the Notch pathway and the JAK/STAT3 signaling pathway. We therefore sought to determine whether the inhibition of the Jagged1/Notch1 signaling pathway can modulate JAK/STAT3 activity. Our western blotting results show that JAK, STAT3, and the phosphorylation of STAT3 (pSTAT3, a marker of activation) were significantly increased at 3 days following SCI but that Jagged1 knockdown significantly reduced this effect compared with that in other groups (Fig. 7a–d). Next, we investigated the role of STAT3 in reactive astrocytes by examining the protein expression levels of GFAP and pSTAT3 in the spinal cord by double immunofluorescence staining at 3 days after SCI. We found that astrocytes displayed a reactive morphology with enlarged soma and processes and GFAP overexpression. The quantification of the GFAP-positive area showed that there was no significant difference among the three groups at 3 days after SCI (Fig. 7e, g). The level of pSTAT3 was undetectable in uninjured spinal cords (data not shown). In the injured spinal cords, we observed pSTAT3 mainly co-localized with reactive astrocytes. The pSTAT3-positive area in Jagged siRNA-injected mice was markedly smaller than that in scramble siRNA-injected mice and control mice. Moreover, Jagged knockdown strongly reduced the percentage of pSTAT3-positive astrocytes (Fig. 7g, h).

## Discussion

In the present study, we investigated the effect of ADSC transplantation on SCI in a mouse model of acute SCI and elucidated the signaling pathways underlying the functional recovery of SCI mice treated with ADSC transplantation. The results of the present study suggest that the administration of ADSCs immediately after contusion SCI can prevent neuroinflammation, promote neural tissue preservation, and eventually promote functional recovery. These neuroprotective effects are at least partially due to the suppression of Jagged1/Notch1 signaling, which subsequently inhibits JAK/STAT3 phosphorylation.

Recent progress in stem cell biology has opened up an avenue for therapeutic strategies that address the problems encountered in SCI, including the loss of neurons, axonal degeneration, demyelination, disrupted vascular structures, and continuous inflammation [40, 41]. ADSCs are now increasingly being considered optimal stem cells for various therapeutic applications because they are easily obtained and because of their self-renewal ability, multipotency, and lack of immunogenicity [42]. Although the in vitro/vivo transdifferentiation potential of ADSCs and the successful application of ADSCs for cell replacement therapy in various neurological diseases, including SCI, have been reported by some groups, the neural differentiation of MSCs in vivo is still controversial [36, 43–45]. We found no evidence in the present study that ADSCs differentiated into glial or neural cells 4 weeks after transplantation into the injured spinal cords of mice. These findings, along with evidence that the transplantation of ADSCs leads to an improvement in locomotor function and a reduction in neural cell death, support the hypothesis that more complex mechanisms than cellular replacement must be responsible for the therapeutic potential of MSCs.

The inflammation process caused by injury to the spinal cord, including the infiltration of macrophages into the injured tissue and the secretion of inflammatory cytokines, results in deleterious neurological deficits at the injury site and in the area adjacent to the lesion and is a key component of the progression of SCI [4]. The immunomodulatory potential of ADSCs to reduce the inflammatory response and improve the microenvironment to promote the survival of both transplanted and endogenous cells has been extensively described [46, 47]. Our findings are in line with these observations. In our present study, by performing immunostaining for ED-1 to label active microglia/macrophages and IBA1 to label all microglia, we observed significant decreases in the numbers of ED-1-positive and IBA1-positive cells upon ADSC transplantation on day 7 after SCI compared to the numbers in the SCI control group. Furthermore, by using q-PCR, we found that ADSC transplantation significantly decreased the expression of proinflammatory cytokines such as IL-6, IL-1β, and TNF-α in the injured spinal cords. The reduction in macroglia/macrophages was associated with enhanced tissue preservation and eventual functional recovery. These results are not surprising because activated infiltrated macroglia/macrophages are believed to secrete more detrimental and proinflammatory cytokines and factors that contribute to neural cell death after SCI. Collectively, our findings support the notion that anti-inflammation is a key mechanism underlying the therapeutic potential of ADSCs in SCI.

The precise mechanism through which MSCs affect the inflammatory reaction following SCI is still unclear. In recent years, Jagged/Notch1 signaling, which is critical for cell fate decisions and endogenous neurogenesis, has been regarded as a potential therapeutic target for modulating the inflammatory response after CNS injury [27, 48]. Preventing the cleavage of Notch1 into the NICD by *N*-[*N*-(3,5-difluorophenacetyl-l-alanyl)]-*S*-phenylglycine t-butyl ester (DAPT, a γ-secretase inhibitor) augments the regeneration of motor neurons in the injured spinal cords of zebrafish [49] and promotes endogenous neurogenesis and axonal reorganization in a rat stroke model [48]. Arumugam et al. further demonstrated that Notch endangers neurons by modulating pathways that increase their vulnerability to apoptosis and that by activating microglial cells and stimulating the infiltration of proinflammatory leukocytes, the suppression of Notch signaling by DAPT reduces brain damage and improves functional outcomes in a mouse model of stroke [21]. These findings indicate that the activation of Notch signaling is responsible for the progression of neuroinflammation. Recently, the inhibitory effects of MSC transplantation on Notch1 signaling in various diseases were confirmed by several groups. For example, MSC transplantation alleviates early brain injury in subarachnoid hemorrhage [27] and improves osteopenia in lupus [26] by suppressing Notch1 signaling. Naturally, we hypothesized that ADSC treatment may alleviate the inflammatory response and neurodegeneration by inhibiting the Jagged1/Notch signaling pathway after SCI.

To evaluate the activation of the Notch signaling pathway after SCI, we used western blotting to detect the expression level of NICD (the activated form of Notch1). Our results showed that NICD was maximally activated 3 days post-injury and that its expression was reversed 7 and 14 days after SCI. The western blotting results were further confirmed by immunofluorescence staining. These results are in accordance with a previous study indicating that Notch1 signaling is activated in the acute phase of SCI and then gradually declines in the subacute stage [50, 51]. Our double immunofluorescence findings showed that NICD was mainly expressed in NeuN-positive neurons. We did not detect NICD in astrocytes, and despite the reported effects of Notch1 in microglia, only very few IBA-positive microglia stained positive for NICD [27, 52, 53]. Jagged1 was found in GFAP-positive astrocytes and NeuN-positive neurons, and very weak Jagged1 immunofluorescence intensity was observed in IBA-positive microglia. Our results indicated that Notch signaling was mainly activated in astrocytes and neurons in the mouse model of SCI used in the present study. Our results are in agreement with several previous studies [22, 54]. The reason for the discrepancy between these studies is unknown, but a possible explanation for the difference in the levels of NICD and Jagged1 in microglia is the different animal strains and animal models used in these studies. Further investigations are needed to explore the exact mechanism. In addition, we found that ADSC administration significantly inhibited the expression of Jagged1/Notch1 and its downstream factors, including Jagged1, NICD, and RBP-JK, compared with that in the SCI control group after SCI. Taken together, our results suggest that ADSC administration immediately after contusion SCI inhibits the activation of the Jagged1/Notch signaling pathway.

To evaluate whether the inhibition of Jagged1/Notch signaling is responsible for the therapeutic potential of ADSCs in SCI mice observed in the present study, we injected a lentivirus containing Jagged1 siRNA into the mouse spinal cord to knock down Jagged1/Notch. Our results show that Jagged1/Notch signaling was significantly suppressed by Jagged1 siRNA at 3 days post-injection. In addition, the expression of proinflammatory cytokines in injured spinal cords was significantly reduced by Jagged1 siRNA injection 3 days post-SCI, which was confirmed by measuring the mRNA levels of IL-6, IL-1β, and TNF-α. We next addressed whether Jagged1 siRNA injection helps maintain neuronal viability. The results of NeuN staining showed that from day 7, the number of viable, non-atrophic neurons with strong NeuN immunostaining was nearly twofold greater than that in the SCI control group and SCI+scramble siRNA-injected group. Neuronal death was confirmed by activated caspase-3/NeuN co-labeling at 7 days post-SCI. The percentage of activated caspase-3/NeuN co-labeling neurons in the ventral horns of SCI control mice and SCI+scramble siRNA-injected mice was almost twice as high as that in the ventral horns of SCI+Jagged1 siRNA-injected mice. These results suggest that Jagged1/Notch inactivation reduces neuroinflammation and promotes neuronal integrity after SCI. In addition, in our in vitro study, co-culture with ADSC reduced the OGD-induced apoptosis and neuroinflammation in cultured neuronal cells; the protective effects of ADSC against apoptosis and inflammation were reduced by treatment with recombinant Jagged1-Fc protein. Taken together, these results indicate that the therapeutic effects of ADSC transplantation in SCI mice observed in the present study were at least partly due to the inhibition of Jagged1/Notch signaling. As mentioned above, the expression of Notch and its downstream factors in microglia/macrophages is weak, and the molecular mechanism by which the inhibition of the Jagged1/Notch pathway downregulates the neuroinflammatory response after SCI needs further investigation.

Astrocytes, as the most abundant cells in the CNS, became reactive in virtually all diseases of the CNS, and this process involves significant transcriptional changes. For example, recent studies have identified at least two types of reactive astrocytes, A1 neurotoxic reactive astrocytes and A2 protective astrocytes [55]. It has been reported that the signaling mechanisms that regulate astrocyte reactivity after SCI are important targets for alleviating neuroinflammation. The Janus kinase/signal transducer and activator of transcription 3 (JAK/Stat3) pathway is a core signaling cascade for the induction and maintenance of astrocyte reactivity [29, 56]. The activation of Stat3 via phosphorylation by Janus kinases (JAKs) has been demonstrated in a variety of disease models, including SCI [57–59]. The inhibition of this pathway mitigates the neuroinflammatory response and restores synaptic deficits [59, 60]. Numerous studies have demonstrated a strong association between Notch and Stat3 signaling, and a change in Notch expression can alter Stat3 phosphorylation and activity [32, 61]. More recently, it was demonstrated that the inhibition of the Notch pathway by DAPT suppresses Stat3 activation in astrocytes in SCI rats, facilitating the transformation of astrocytes to the A1 neurotoxic reactive astrocyte phenotype and ultimately mitigating inflammation-associated secondary damage following SCI [50]. These studies suggest that Notch signaling can alter the astrocyte phenotype by affecting Stat3 activity and function. In the present study, we explored whether Jagged1/Notch knockdown reduced the activation of JAK/STAT3 in the spinal cord. Our findings suggested that the protein levels of JAK, STAT3, and p-STAT3 were significantly reduced after Jagged1 knockdown 3 days after SCI. Furthermore, by using double immunofluorescence assays, we found that Jagged1/Notch knockdown significantly reduced the percentage of p-STAT3-positive astrocytes compared to that in the control SCI mice. Based on these results, we speculate that Jagged1/Notch signaling may participate in the neuroinflammatory response in SCI by mediating the phosphorylation of the JAK/STAT3 signaling pathway in astrocytes.

## Conclusion

Here, we enhance the understanding of ADSC-based cellular therapy by providing experimental evidence that the neuroprotective effects of ADSCs in the treatment of SCI are associated with Jagged1/Notch signaling. ADSC treatment suppresses the activation of the Jagged1/Notch pathway after SCI, which inhibits the phosphorylation of JAK/STAT3 and then alleviates the neuroinflammatory response.

## Supplementary information

**Additional file 1: Table S1.** The number of mice in each 8 experimental group of the experiments part 1. **Table S2.** The number of mice in each experimental group of the experiments in part 2.

**Additional file 2: Figure S1.** The effect of Jagged1/Notch pathway on inflammation and apoptosis in ADSC co-cultured with neuronal cells.

## Data Availability

All data generated or analyzed during this study are included in this published article.

## Abbreviations

MSCs: Mesenchymal stem cells
ADSC: Adipose mesenchymal stem cell
GFP: Green fluorescent protein
IBA1: Ionized calcium-binding adapter molecule 1
JAK/STAT3: Janus tyrosine kinase/signal transducer and activator of transcription
SCI: Spinal cord injury
CNS: Central nervous system
NICD: Notch intracellular domain
GFAP: Glial fibrillary acidic protein
TNF-α: Tumor necrosis factor alpha
IL: Interleukin
ANOVA: Analysis of variance
siRNA: Small interfering RNA
RBP-JK: Recombining binding protein suppressor of hairless
BMS: Basso Mouse Scale
