# Supplementary material for: Adipose mesenchymal stem cell transplantation alleviates spinal cord injury-induced neuroinflammation partly by suppressing the Jagged1/Notch pathway
Source: Stem Cell Res Ther. 2020 Jun 3;11:212. doi: 10.1186/s13287-020-01724-5 (PMC7268310; doi:10.1186/s13287-020-01724-5)
Supplement: Supplementary file 1 — Additional file 1: Table S1. The number of mice in each 8 experimental group of the experiments part 1. Table S2. The number of mice in each experimental group of the experiments in part 2. [file 13287_2020_1724_MOESM1_ESM.docx]

Table 1. The number of mice in each experimental group of the experiments part 1.

| **Survival time Analysis Animal group**  **Sham Control ADSC** |
| --- |
| 1 day NICD WB/immunoreactivity 4/4 4/4 4/4  3 days NICD immunoreactivity - 4 4  NICD/Jagged1/RBP-JK WB 4 4 4  IL-6/TNF-α/IL-1β Q-PCR 5 5 5  7 days NICD WB/immunoreactivity - 4/4 4/4  Double immunofluorescence - 4 -  14 days NICD WB/immunoreactivity - 4/4 4/4  21days NICD WB/immunoreactivity - 4/4 4/4  28 days Cell survival/differentiation 3 3 3  H&E staining/Double immunofluorescence  4 4 4 |

The number of mice in each group in this part of the experiment is provided.

Table 2. The number of mice in each experimental group of the experiments in part 2.

| **Survival time Analysis Animal group**  **Sham Control SCI+ SCI+ scramble siRNA Jagged1 siRNA** |
| --- |
| 3 days NICD/Jagged1/RBP-JK 4 4 4 4  and JAK/STAT3/pSTAT3 WB  IL-6/TNF-α/IL-1β Q-PCR 5 5 5 5  NeuN immunoreactivity 4 4 4 4  Double immunofluorescence 4 4 4 4  (Activated caspase-3/NeuN + pSTAT3/GFAP)  7 days NeuN immunoreactivity - 4 4 4  14 days NeuN immunoreactivity - 4 4 4  28 days NeuN immunoreactivity - 4 4 4 |
