# Supplementary material for: Adipose mesenchymal stem cell transplantation alleviates spinal cord injury-induced neuroinflammation partly by suppressing the Jagged1/Notch pathway
Source: Stem Cell Res Ther. 2020 Jun 3;11:212. doi: 10.1186/s13287-020-01724-5 (PMC7268310; doi:10.1186/s13287-020-01724-5)
Supplement: Supplementary file 2 — Additional file 2: Figure S1. The effect of Jagged1/Notch pathway on inflammation and apoptosis in ADSC co-cultured with neuronal cells. [file 13287_2020_1724_MOESM2_ESM.zip › additional file 2.docx]

**Supplementary Fig.1 The effect of Jagged1/Notch pathway on inflammation and apoptosis in ADSC co-cultured with neuronal cells.**

ELISA results indicating the levels of the pro-inflammatory factors TNF-α and IL-1β in the OGD-injured neurons was significantly enhanced compared with those in non-OGD exposed cells (A, B) (P<0.05). Co-culturing OGD-injured neurons with ADSC decreased the expression of TNF-α and IL-1β. Jagged1 siRNA treatment effectively enhanced the ADSC co-culture induced suppression of the expression of TNF-α and IL-1β, whereas Jagged-1 (1μg/ml, 24 h) treatment abolished the suppressive effects of ADSC co-culture. (*P < 0.05 versus the non-OGD injured neurons; #P <0.05 versus the neurons co-culture with ADSC; &P <0.05 versus OGD-injured neurons). Representative image of Western blotting (C, D) and quantification (E, F) of activated caspase3 in neurons following different treatments. (*P < 0.05 versus the non-OGD injured neurons; #P <0.05 versus the neurons co-culture with ADSC; &P <0.05 versus OGD-injured neurons).
