# Supplementary figures and images for: Adipose mesenchymal stem cell transplantation alleviates spinal cord injury-induced neuroinflammation partly by suppressing the Jagged1/Notch pathway
Source: Stem Cell Res Ther. 2020 Jun 3;11:212. doi: 10.1186/s13287-020-01724-5 (PMC7268310; doi:10.1186/s13287-020-01724-5)

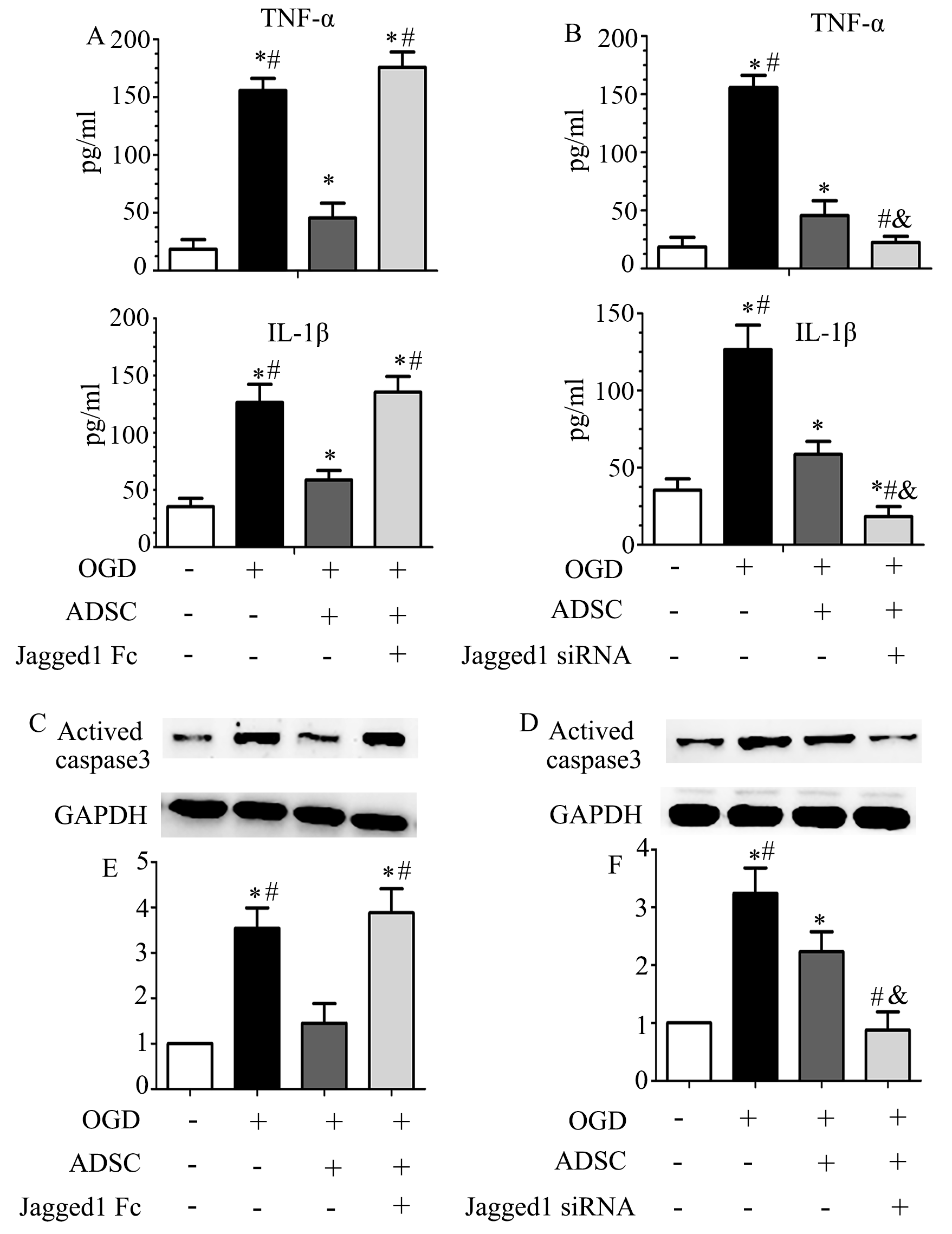

Supplement: Supplementary file 2 — Additional file 2: Figure S1. The effect of Jagged1/Notch pathway on inflammation and apoptosis in ADSC co-cultured with neuronal cells. [file 13287_2020_1724_MOESM2_ESM.zip › Supplementary Fig. 1.tif]
